# Prospective ultrasonographic evaluation of femoral and vastus intermedius muscles as predictors of ICU-acquired weakness in critically ill patients

**DOI:** 10.1007/s40477-025-01013-y

**Published:** 2025-04-22

**Authors:** A. M. Chaves, S. J. Torres, L. Palacios, JI Alvarado, M. V. Stozitzky, C. A. Santacruz H

**Affiliations:** 1 Department of Intensive and Critical Care Medicine, Academic Hospital Fundación Santa Fe de Bogotá, Bogotá, Colombia; 2 School of Medicine, Universidad del Rosario, Bogotá, Colombia; 3 Instituto de Medicina del Ejercicio y Rehabilitación (IMER), Academic Hospital Fundación Santa Fe de Bogotá, Bogotá, Colombia

**Keywords:** Intensive care unit acquired weakness (ICU-AW), Medical Research Council score (MRC), Sequential Organ Failure Assessment Score (SOFA), Acute Physiology and Chronic Health Evaluation (APACHE), Muscle ultrasound (MUS), Femoral cross-sectional area (Fcsa), Femoral + vastus intermedius thickness (F + VIth)

## Abstract

**Purpose:**

Intensive care unit-acquired weakness (ICU-AW) is associated with poor functional outcomes and increased healthcare costs. This study aimed to evaluate the diagnostic performance of muscular ultrasound (MUS) measurements in predicting ICU-AW and identify potential predictors.

**Methods:**

Forty-three surgical and medical ICU patients underwent serial MUS measurements of the femoral cross-sectional area (Fcsa) and femoral + vastus intermedius thickness (F + VIth) on days 1, 3 and 5 post-ICU admission. Patients were categorized as having ICU-AW (Medical Research Council (MRC) sum score < 48 at discharge) or not. Univariate and multivariate logistic regression analyses were performed to identify predictors of ICU-AW. The diagnostic performance of MUS measurements was assessed via receiver operating characteristic (ROC) curves. Clinical outcomes (ICU length of stay, ventilator days, extubation failure) were compared between the groups.

**Results:**

Patients with ICU-AW (*n* = 12, 28%) showed a significant reduction in the Fcsa from Day 1 to Day 5 (*p* < 0.001). Univariate analysis revealed significant associations between ICU-AW and the Apache II score (OR 1.12, *p* = 0.03), SOFA score (OR 1.32, *p* = 0.008), and Day 1 F + VIth score (OR 0.23, *p* = 0.05). Multivariate analysis confirmed a significant association with the SOFA score (OR 1.35, *p* = 0.04) and a trend toward an F + VIth score of Day 1 (OR 0.12, *p* = 0.09). The day 1 Fcsa and F + VIth demonstrated moderate predictive capabilities for ICU-AW (ROC-AUC values of 0.72 and 0.82, respectively). ICU-AW patients experienced longer ICU stays, more ventilator days, and higher extubation failure rates.

**Conclusion:**

Preexisting low muscle mass, combined with a high SOFA score, may be a stronger predictor of ICU-acquired weakness than the degree of subsequent muscle loss.

**Supplementary Information:**

The online version contains supplementary material available at 10.1007/s40477-025-01013-y.

## Introduction

Intensive care unit-acquired weakness (ICU-AW) is a prevalent and debilitating condition that results in significant muscle wasting and long-term disability in critically ill patients [1]. It affects 20–60% of ICU admissions and is associated with increased mortality rates, prolonged ventilation, the need for tracheostomy, and extended ICU length of stay (LOS) [2]. Given that muscle loss occurs rapidly after admission, early diagnosis and prediction are essential for improving patient outcomes [3].

The Medical Research Council (MRC) score currently serves as the standard for ICU-AW diagnosis [4, 5]; however, its reliance on conscious patients limits its application in those unable to cooperate. Alternative methods, such as risk prediction models and severity score-based methods such as the APACHE and SOFA, have been developed [6–8]. Despite their utility, the APACHE-II score primarily assesses admission severity, whereas the SOFA score tracks organ dysfunction but lacks specificity for early muscle function changes. Moreover, these methods often involve complex computations and lack external validation, which can prolong ICU stays [9].

Recent studies have highlighted muscular ultrasonography as a promising tool for evaluating muscle characteristics in critically ill patients. Ultrasound measurement of the femoral cross-sectional area (Fcsa) and vastus intermedius thickness (F + VIth) not only allows real-time visualization but also enables the serial monitoring of muscle wasting, facilitating early detection—even in sedated patients. Notably, changes in the rectus femoris cross-sectional area and pennation angle have been shown to significantly predict ICU-AW before patients can perform volitional tests [9]. Furthermore, ultrasonographic assessment of the diaphragm and paravertebral intercostals indicates alterations in peripheral and respiratory muscles within the first week of mechanical ventilation, reflecting the need for continuous monitoring [10].

This study hypothesizes that early femoral muscle ultrasound effectively predicts ICU-AW in mechanically ventilated patients. We aimed to evaluate the discriminatory capacity of Fcsa and F + VIth ultrasound measurements for predicting ICU-AW and their associations with ICU LOS, mechanical ventilation (MV) days, tracheostomy needs, and ICU mortality.

## Materials and methods

### Principal and secondary outcomes

The main aim of this study was to assess the ability of the Fcsa and F + VIth MUS to predict ICU-AW. Other outcomes included the associations between Fcsa and F + VIth MUS and the outcomes of (MV) days, (ICU-LOS), extubation failure, need for tracheostomy, and ICU mortality.

### Design and ethical approval

This cross-sectional observational study was performed from June 2022 to January 2023 in the mixed medical–surgical ICU of the Academic Hospital Fundación Santa Fe de Bogotá, Colombia. The Institutional Review Board approved the study (CCEI-13851–2022). Given that the study was observational following current clinical practices, the requirement for written consent to participate was waived by the ethics committee.

### Inclusion and exclusion criteria

The inclusion criterion for this study was that the participants were representative of the general population of critically ill patients at risk for ICUAW. The inclusion criteria for patients were age 18–75 years with potentially reversible pathologies, an expected stay of more than 3 days, and a body mass index below 30 (BMI <30). We excluded patients with any known neuromuscular disorders or recent spinal cord injuries because these conditions are likely to confound the results related to muscle strength and ultrasound measurements. Neuromuscular disorders often lead to chronic muscle weakness and may not be solely attributable to critical illness, potentially skewing our findings regarding ICU-AW. We also excluded patients whose (BMI) was greater than 30, as obesity can complicate ultrasound measurements and introduce variability in muscle thickness and cross-sectional assessments. We also excluded patients for whom no legs were available for muscle strength testing or ultrasound, patients with acute or chronic immobilization prior to ICU admission, and patients with soft tissue injuries that would prevent measurement of the muscle thickness and/or cross-sectional area.

### Clinical data collection

We collected the following clinical characteristics into a predefined table: age, sex, body weight and length at ICU stay; admission type; admission diagnosis; Acute Physiology and Chronic Health Evaluation II (APACHE II) score; maximal total Sequential Organ Failure Assessment (SOFA) score; presence of sepsis (according to the Sepsis-3 criteria [11]), 1and laboratory data, including albumin, total protein, triglyceride, and cholesterol levels, before inclusion. In addition, we collected data on preexisting polyneuropathy or myopathy, risk factors for polyneuropathy before ICU admission (diabetes mellitus, alcohol abuse, chemotherapy, and kidney failure), ICU length of stay (ICU-LOS), mechanical ventilation days, extubation failure, need for tracheostomy, MRC Day 1 and Day 5, and ICU mortality.

### Measurements

#### Medical Research Council score (MRC) (the reference standard)

Muscle strength was assessed as soon as patients were awake [Richmond Agitation Sedation Scale (RASS) score between − 1 and 1] and cooperative. Assessment was performed by a trained and experienced physiotherapist who was blinded to the ultrasound results. The MRC score was used to assess strength in the following six muscle groups bilaterally: wrist dorsiflexors, elbow flexors, shoulder abductors, hip flexors, knee extensors and ankle dorsiflexors. Scores ranging from 0 to 60 indicate normal strength in all four limbs. ICU-AW was defined as a mean MRC sum score < 48, in accordance with the international consensus statement [12].

#### Muscular ultrasound (MUS) measurements (the index test)

The ultrasound measurements (thickness of the rectus femoris muscle plus the vastus intermedius and transverse cross-sectional area of the rectus femoris muscle) were performed via a Mindray ultrasound machine, model TE7, and linear probe L14-6 N (6–14 megahertz). The B (brightness) mode was used in the muscular preset for the two previously mentioned measurements, and the data obtained were recorded in the collection instrument. All ultrasound machines used in this study were calibrated according to the manufacturer's recommendations to ensure consistent and accurate measurements.

The ultrasound muscle measurement protocol for the transverse cross-sectional area of the rectus femoris (Fcsa) and the thickness of the rectus femoris plus the vastus intermedius (F + VIth) were adjusted in a proprietary way and based on previously published reports [9, 10], with the goal of achieving the same measurements performed on different days in the ICU at the same location as the one performed initially.

We started with proper positioning of the patient, which consisted of total extension of the lower extremities, maintaining a 0-degree angle at the knees of the patient and the tips of the fingers pointing toward the ceiling. The angle formed by the hip was considered indifferent; however, for this research, the angle was 30–45 degrees according to the base position of the patient's headboard. With a tape measure on the selected lower extremity, the distance in centimeters was subsequently measured from the greater trochanter of the femur to the superior edge of the patella. This distance was divided by 3, and the resulting value in centimeters was measured from the superior edge of the patella toward the cranial direction (point corresponding to the beginning of the lower third of the quadriceps femoris) and marked to define the exact point where subsequent measurements were performed. The transducer was then positioned in a transverse axis with respect to the extremity at the level of the previously marked point, forming a 90-degree angle with respect to the extremity exerting minimal pressure with the transducer. At this level, the fascia of the rectus femoris muscle can also be identified, which limits measurement of the transverse area of the rectus femoris. Later, the thickness of the rectus femoris and vastus intermedius was measured by calculating the distance from the superior fascia of the rectus femoris to the femur epiphysis. Intra- and interobserver reliability were assessed by calculating the intraclass correlation coefficient (ICC). Three pairs of measurements were carried out in the right limb in a protocolized manner at the convenience site.

#### Study protocol

Clinical variables and MUS measurements were evaluated at ICU admission (day 1). MUS measurements were subsequently performed again on day 3 and day 5. The MRC scores were obtained specifically at ICU discharge.

### Statistical analysis

The data are presented in terms of proportions (percentages), means (standard deviations), or medians (25th to 75th percentiles), as appropriate. ICU-AW was defined as a mean MRC sum score < 48. The normality of the distribution was assessed via the Shapiro‒Wilk test. Statistical significance was assessed via parametric and non-parametric tests as appropriate. Differences between times were evaluated via the Kruskal‒Wallis test, with post hoc analysis conducted via the Bonferroni correction. The discriminative power of MUS for Day 1 to Day 5 measurements, absolute (ΔAbs) and proportional (Δ%) changes in F + VIth and Fcsa, and the "optimal cutoff point" for ICU-AW development were determined via receiver operating characteristic area under the curve (ROC-AUC) with 95% confidence intervals. The Youden test was used to identify the optimal cutoff point, and the Delong test was used to evaluate differences in the areas under the curves (AUCs). The discriminative power of AUC values between 90 and 100% was defined as excellent, between 80 and 89% as good, between 70 and 79% as moderate, between 60 and 69% as poor and < 60% as failed. The analysis was performed via R statistical software (latest version), and statistical significance was set at a *p* value < 0.05 [13–15].

## Results

A total of 76 patients were screened, and a total of 43 patients were included, 12 (28%) of whom developed ICU-AW at ICU discharge (Table 1). Considering all the measurements (*n* = 20), the median difference between the two measurements for the Fcsa was 0.09 cm [95% CI, – 0.06 to – 0.115], and the calculated ICC was 0.98 [95% CI 0.92 to – 0.99].The median difference between the two measurements for F + VIth was – 0.005 cm [95% CI – 0.095 to 0.02], and the calculated ICC was 0.98 [95% CI 0.92 to – 0.99].

**Table 1:** Baseline characteristics of included patients. Data are expressed as percentages (%) or medians [IQR] as appropriate

Characteristic	ICU-acquired weakness		*p* value^2^
	No, *N* = 31^1^	Yes, *N* = 12^1^	
Age, years	55 [41- 64]	66 [50–74]	0.2
Sex			
Female	12 (39%)	5 (42%)	> 0.9
Male	19 (61%)	7 (58%)	> 0.9
Weight, Kg	70 [62–80]	67 [60–70]	0.3
Height, mt	1.63 [1.59–1.71]	1.65 [1.60–1.67]	> 0.9
BMI	26 [22–27]	23 [22–26]	0.3
APACHE score	8 [5–10]	12 [10–17]	0.008
SOFA score	4 [1–6]	7 [6–9]	0.004
Type of Diagnosis, *n* (%)			
Burn	8 (26%)	0 (0%)	NA
Medical	0 (0%)	1 (8%)	0.33
Neurological	5 (16%)	3 (25%)	0.43
Respiratory	1 (3%)	3 (25%)	0.42
Sepsis	3 (10%)	2 (17%)	0.28
Surgery	14 (45%)	3 (25%)	0.12
Laboratory			
Creatinine, mg/dL	0.95 [0.74–1.20]	0.88 [0.70–0.95]	0.2
Vitamin D3, IU	22 [18–26]	17 [12–18]	0.03
Hemoglobin, gr/dL	13.5 [12.1- 15.0]	10.2 [8.0- 12.8]	0.01
Albumin, g/dL	3.4 [3.1- 3.8]	2.8 [2.3- 3.4]	0.06
Pre-albumin, mg/dL	19 [16–24]	13 [10–17]	0.04
Triglycerides, mg/dL	131 [91–188]	127 [97–166]	> 0.9
Cholesterol, mg/dL	127 [114–163]	103 [84–109]	0.02
Outcome			
ICU-LOS, days	6 [5–10]	14 [8–22]	0.005
Mechanical ventilation days	2 [1–5]	7 [4–20]	0.003
Extubation failure, %	1 (5.0%)	4 (40%)	0.03
MRC discharge	58 [52–60]	40 [32–45]	< 0.001

*ICU-LOS* intensive care unit length of stay, *MRC* medical research council

**Fig. 1:**
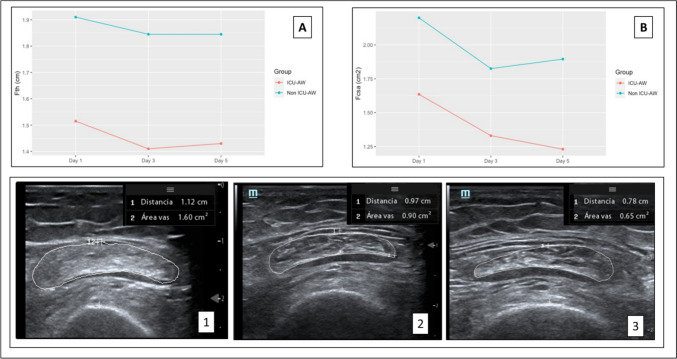
Evolution of ultrasonographic longitudinal changes in rectus femoris cross-sectional area (Fcsa) and the combined thickness of rectus femoris and vastus intermedius (F + VIth) muscles, measured via ultrasound on days 1, 3, and 5 post-ICU admission. **A** Evolutionary trend of Fth in ICU-AW vs. non-ICU-AW groups. **B** Evolutionary trend of Fcsa in ICU-AW and non-ICU-AW groups. In the bottom: Evolutionary trend of the two measurements (Fcsa and Fth) in ultrasound images of a patient on admission (Measure 1), day 3 (Measure 2), and day 5 (Measure 3) of ICU stay. Measurements were obtained using a standardized protocol using a linear transducer with measurements taken at the lower third of the quadriceps femoris. The transducer was then positioned in a transverse axis with respect to the extremity at the level of the previously marked point, forming a 90-degree angle with respect to the extremity. Potential confounding factors such as pre-existing muscle conditions were excluded (see text for complete list of exclusion criteria)

At ICU admission, patients who developed ICU-AW presented a decrease in F + VIth (1.52 cm [1.26–1.60] vs. 1.91 [1.66–2.31]; *p* < 0.001) and Fcsa (1.64 cm^2^ [1.46–1.97] vs. 2.20 [1.78–2.68]; *p* = 0.02) (Supplemental Table S1). Additionally, patients with ICU-AW experienced a reduction in F + VI values (– 5.92%; 95% CI [– 10.31%, – 1.25%] vs – 3.13%; 95% CI [– 7.21%, 1.27%]) and in Fcsa values (− 24.94%; 95% CI [– 30.12% to – 19.12%] vs – 13.64%; 95% CI [– 18.42%, – 8.23%]) from Day 1 to Day 5 (Fig. 1).

**Fig. 2:**
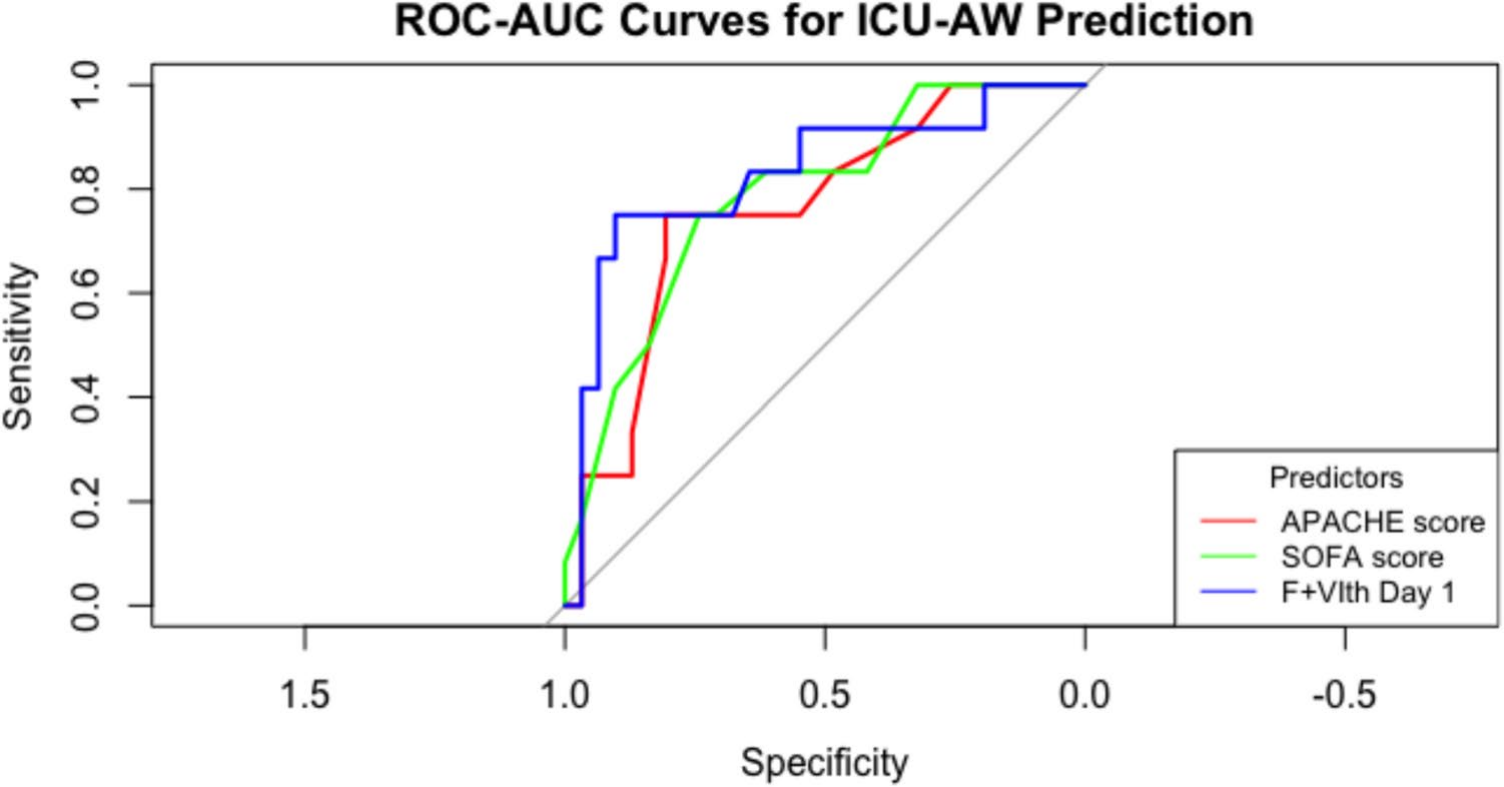
ROC-AUC plot for predictor with the highest AUC (> 0.8) as compared to APACHE and SOFA scores. *APACHE* Acute Physiologic and Chronic Health Evaluation, *SOFA* Sequential Organ Failure Assessment, *F + VIth* femoral + vastus intermedius muscle

Univariate analysis revealed predictors with significant odds ratios: APACHE score OR 1.12; *p* = 0.03; SOFA score OR 1.32; *p* = 0.008; and F + VIth Day 1 OR 0.23; *p* = 0.05. Multivariate analysis revealed a significant relationship between ICU-AW and the SOFA score (OR 1.35; *p* = 0.04) and a trend toward F + VIth day 1 (OR 0.12; *p* = 0.09) (Supplemental Appendix Table S2). The day 1 F + VIth AUC-ROC (0.83, 95% CI [0.66–0.95; best cutoff point 1.58 cm; sensitivity 75%, specificity 90%), APACHE II (0.76, 95% CI [0.60–0.90] best cutoff point 10; sensitivity 75%, specificity 80%) (DeLong for difference between ROC-AUC *p* value = 0.36) and SOFA (0.78, 95% CI [0.61–0.91] (the best cutoff point 6; sensitivity 75%, specificity 74%) (DeLong for difference between ROC-AUC: *p* value = 0.67) scores showed the highest discriminatory capacities for ICU-AW prediction (Fig. 2). Patients with ICU-AW (28%) experienced prolonged ICU-LOS (14 days [8–22] vs. 6 [5–10]; *p* = 0.005), increased MV days (7 days [4–20] vs. 2 [1–5]; *p* = 0.003), and increased rates of extubation failure (40% vs. 5%; *p* = 0.03) (Supplemental Appendix Fig. S1). The laboratory risk factors for ICU-AW included low vitamin D3 (17 [12–18] vs. 22 [18–26] ng/mL; *p* = 0.03), prealbumin (13 [10–17] vs. 19 [16–24] mg/dL; *p* = 0.04), and cholesterol (103 [84–109] vs. 127 [114–163] mg/dL) levels.

## Discussion

Low femoral and vastus intermedius muscle thickness at ICU admission predicted (ICU-AW). While both groups showed muscle mass reduction, the decline was greater and more prolonged in ICU-AW patients. Consistent with prior studies [3, 10], we observed decreased muscle mass (cross-sectional area and thickness), along with laboratory evidence of protein and caloric malnutrition. This reduction was more pronounced in patients with higher SOFA and APACHE scores and lower serum protein levels, indicating increased catabolism typical of critical illness [16]. Importantly, we observed differences in (MRC) scores between ICU-AW patients and non-ICU-AW patients, minimizing selection bias and ensuring that muscle mass changes reflected true ICU-AW, not other causes of muscle wasting [3, 10, 17]. Because MRC scores were assessed at ICU discharge, our results suggest that (MUS) may moderately predict ICU-AW development. MUS may prove as valuable a prognostic tool as the APACHE and SOFA scores for predicting ICU-AW [7, 16, 18]. Early MUS assessment could guide preventive measures and treatment, particularly in sedated patients, where standard scales are less effective.

 (ICU-AW) arises from a complex interplay of factors leading to skeletal muscle dysfunction and atrophy [17]. The hallmarks of ICU-AW are an inflammatory response, bioenergetic dysfunction, altered protein balance, neuronal axon degeneration, changes in muscle histology, and muscle wasting [18]. Muscle damage is often mediated by inflammatory cytokines, microcirculatory disturbances that reduce the oxygen supply, bioenergetic mitochondria impairment that causes reduced ATP production, and disruptions in the ion channel membrane [19]. Sepsis exacerbates this process through the release of proinflammatory mediators that impair cellular function and protein synthesis within muscle tissue [19]. Consequently, patients with high muscle loss have higher organ dysfunction scores [19], longer stays in the ICU, and higher ICU and hospital mortality [20–24]. Moreover, disuse atrophy, stemming from prolonged immobility and reduced neuromuscular activity common in critically ill patients, contributes significantly to muscle mass loss [18]. This reduced activity diminishes protein synthesis while increasing protein breakdown, further accelerating muscle wasting.

The MRC sum score, while the established reference standard for ICU-AW, presents limitations that warrant consideration. Its reliance on patient cooperation and volitional effort restricts its utility in the early stages of critical illness, particularly in sedated or mechanically ventilated patients, a substantial proportion of the ICU population [25]. This inability to assess muscle strength in these patients represents a significant diagnostic gap. The objective, quantitative data provided by ultrasound, regardless of patient sedation status, offer a crucial advantage in addressing this limitation. The noninvasive nature of ultrasound allows for serial monitoring, enabling early detection of muscle changes and facilitating timely interventions before significant muscle wasting occurs [9, 10]. While the MRC sum score remains a valuable tool in appropriate clinical settings, our study highlights the potential of ultrasound as a complementary approach for the early diagnosis and management of ICU-AW, particularly in critically ill sedated ICU patients.

The early detection afforded by ultrasound may allow clinicians to intervene before these processes cause irreversible damage [25, 26]. Our findings suggest that early identification of patients at high risk for developing ICU-AW via ultrasound may be as good as the use of more established scores such as the SOFA and APACHE II scores. Moreover, an F + VIth cutoff of 1.58 cm yielded moderate sensitivity and high specificity, suggesting that this noninvasive, readily available assessment could be used [27]. This early identification has possible clinical implications, enabling timely implementation of preventative strategies. For example, a lower F + VIth value, indicating potential for ICU-AW, could prompt earlier initiation of interventions such as targeted nutrition [26], proactive physiotherapy (including early mobilization), and/or respiratory muscle training [28]. These proactive interventions could decrease the severity of muscle wasting, shorten recovery times, and ultimately improve functional outcomes for ICU survivors. The high specificity (90%) may also minimize the risk of unnecessary interventions for patients not at risk of ICU-AW.

Our study had several limitations. We did not normalize the raw ultrasound data to normal values from healthy individuals. As such, the data from our study may have been biased since F + VIth and Fcsa can be age-, sex-, dominance-, length-, or weight-dependent [27, 29]. We did not perform electromyography in patients with ICU-AW, so we cannot readily distinguish between muscle and/or nerve involvement (e.g., critical illness polyneuropathy) [30]. While our study acknowledges the potential for selection bias due to the unequal distribution of neurological and post-surgical patients in the ICU-AW group, subgroup analyses revealed no statistically significant differences in outcomes between these groups.

## Conclusion

Our findings demonstrate that early ultrasound measurement of muscle thickness, combined with clinical parameters such as SOFA scores and indicators of nutritional status, offers a moderate but valuable predictive capacity for ICU-AW. Preexisting low muscle mass appears to be a particularly strong risk factor. While these results are promising, further validation in larger, multicenter studies is crucial to confirm these findings and establish the generalizability of our approach across diverse patient populations and ICU settings. The potential clinical implications are substantial: integrating ultrasound as a routine monitoring tool in at-risk ICU patients could facilitate timely intervention, potentially mitigating the severity and long-term consequences of ICU-AW. Early detection, enabled by readily available ultrasound, allows for the implementation of preventive strategies such as nutritional support and early mobilization, ultimately aiming to improve patient outcomes and reduce the burden of ICUAW.

## Supplementary Information

Below is the link to the electronic supplementary material.

Supplementary file1 (DOCX 43 KB)

Supplementary file2 (DOCX 19 KB)

Supplementary file3 (DOCX 17 KB)

## Data Availability

The datasets generated and/or analyzed during the current study are not publicly available owing to institutional privacy policies but are available from the corresponding author upon reasonable request.
