# Supplementary material for: Prospective ultrasonographic evaluation of femoral and vastus intermedius muscles as predictors of ICU-acquired weakness in critically ill patients
Source: J Ultrasound. 2025 Apr 22;28(2):447–54. doi: 10.1007/s40477-025-01013-y (PMC12145331; doi:10.1007/s40477-025-01013-y)
Supplement: Supplementary file 1 — Supplementary file1 (DOCX 43 KB) [file 40477_2025_1013_MOESM1_ESM.docx]

Supplemental Appendix

Chaves et al.


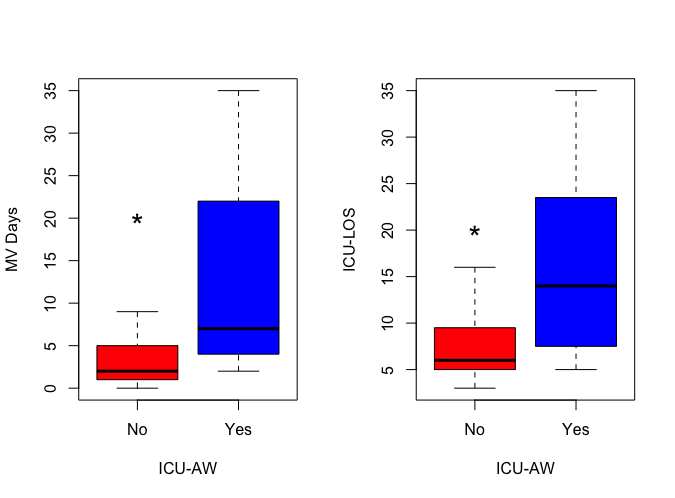


**Supplemental Figure S1. Comparison of Mechanical Ventilation Days (MV Days) and ICU Length of Stay (ICU-LOS) between patients with and without ICU-Acquired Weakness (ICU-AW).** Boxplots illustrate the distribution of MV Days (left panel) and ICU-LOS (right panel) for patients with (Yes) and without (No) ICU-AW. Error bars represent the interquartile range, with whiskers extending to 1.5 times the interquartile range. Outliers are represented by individual points. p<0.05, indicated by *
