# Supplementary material for: Prospective ultrasonographic evaluation of femoral and vastus intermedius muscles as predictors of ICU-acquired weakness in critically ill patients
Source: J Ultrasound. 2025 Apr 22;28(2):447–54. doi: 10.1007/s40477-025-01013-y (PMC12145331; doi:10.1007/s40477-025-01013-y)
Supplement: Supplementary file 2 — Supplementary file2 (DOCX 19 KB) [file 40477_2025_1013_MOESM2_ESM.docx]

Supplemental Apendix

Chaves et al.

|  |  | **ICU-acquired weakness** | |  |
| --- | --- | --- | --- | --- |
| **Characteristic** | **N** | **No**, N = 31^1^ | **Yes**, N = 12^1^ | **p-value**^2^ |
| F+VIth day 1, cm | 43 | 1.91 [1.66 to 2.31] | 1.52 [1.26 to 1.60] | <0.001 |
| F+VIth day 3, cm | 40 | 1.85 [1.47 to 2.19] | 1.41 [1.23 to 1.48] | 0.004 |
| F+VIth day 5, cm | 25 | 1.85 [1.41 to 2.50] | 1.43 [1.30 to 1.55] | 0.08 |
| %Δ F+VIth day 1 vs 3 | 40 | -0.07 [-0.18 to 0.00] | -0.07 [-0.13 to 0.00] | >0.9 |
| %Δ F+VIth day 1 vs 5 | 25 | -0.12 [-0.28 to 0.08] | -0.06 [-0.14 to 0.08] | >0.9 |
| Δ Abs F+VIth day 1 vs 3 | 40 | -0.16 [-0.31 to 0.01] | -0.11 [-0.15 to -0.01] | 0.6 |
| Δ Abs F+VIth day 1 vs 5 | 25 | -0.23 [-0.47 to 0.20] | -0.11 [-0.22 to 0.14] | >0.9 |
| Fcsa day 1, cm^2^ | 43 | 2.20 [1.78 to 2.68] | 1.64 [1.46 to 1.97] | 0.02 |
| Fcsa day 3, cm^2^ | 40 | 1.83 [1.32 to 2.54] | 1.33 [1.14 to 1.47] | 0.02 |
| Fcsa day 5, cm^2^ | 25 | 1.90 [1.54 to 2.55] | 1.23 [0.94 to 1.46] | 0.02 |
| %Δ Fcsa day 1 vs 3 | 40 | -0.18 [-0.3 to 0.01] | -0.21 [-0.42 to -0.11] | 0.4 |
| %Δ Fcsa day 1 vs 5 | 25 | -0.27 [-0.38 to 0.07] | -0.42 [-0.45 to -0.19] | 0.3 |
| Δ Abs Day 3 | 40 | -0.33 [-0.68 to -0.02] | -0.29 [-0.83 to -0.18] | 0.6 |
| Δ Abs Day 5 | 25 | -0.57 [-0.79 to 0.19] | -0.59 [-0.94 to -0.31] | 0.6 |
| ^1^Median [IQR] | | | | |
| ^2^Wilcoxon rank sum test; Wilcoxon rank sum exact test | | | | |

**Supplemental Table 1: Comparison of ultrasonographic measurements between patients with and without ICU-acquired weakness**. AbsΔ: Absolute delta; %Δ: Percentage delta ; Fcsa :Femoral cross-sectional area day; F+Vith: femoral + vastus intermedius thickness.
