# Supplementary material for: Prospective ultrasonographic evaluation of femoral and vastus intermedius muscles as predictors of ICU-acquired weakness in critically ill patients
Source: J Ultrasound. 2025 Apr 22;28(2):447–54. doi: 10.1007/s40477-025-01013-y (PMC12145331; doi:10.1007/s40477-025-01013-y)
Supplement: Supplementary file 3 — Supplementary file3 (DOCX 17 KB) [file 40477_2025_1013_MOESM3_ESM.docx]

Supplemental Appendix

Chaves et al.

| *Univariate logistic regression* | | | | | | |
| --- | --- | --- | --- | --- | --- | --- |
| Predictor | *Odds Ratio* | *Lower CI* | | | *Upper CI* | *P-value* |
| SOFA score | 1.32 | 1.07 | | | 1.63 | 0.008 |
| APACHE score | 1.12 | 1.01 | | | 1.24 | 0.03 |
| F+Vith, cm | 0.23 | 0.05 | | | 1.02 | 0.05 |
| Fcsa, cm^2^ | 0.59 | 0.21 | | | 1.65 | 0.31 |
| *Multivariate Logistic Regression Results* | | | | | | |
| SOFA score | 1.34 | | 1.00 | 1.81 | | 0.04 |
| APACHE score | 0.97 | | 0.82 | 1.14 | | 0.77 |
| F+Vith, cm | 0.12 | | 0.01 | 1.43 | | 0.09 |
| Fcsa, cm^2^ | 2.3 | | 0.41 | 12.87 | | 0.34 |

**Supplemental Table S2: Univariate and multivariate logistic regression analyses of predictors for ICU-acquired weakness.** Abbreviations: ICU, Intensive Care Unit; IAW, ICU-acquired weakness; OR, Odds Ratio; CI, Confidence Interval; SOFA, Sequential Organ Failure Assessment; Apache II, Acute Physiology and Chronic Health Evaluation II. Fcsa: Femoral cross-sectional area day; F+VIth: femoral + vastus intermedius thickness.
